# The High Obesity Program: Overview of the Centers for Disease Control and Prevention and Cooperative Extension Services Efforts to Address Obesity

**DOI:** 10.5888/pcd17.190235

**Published:** 2020-03-19

**Authors:** Ashleigh L. Murriel, Sahra Kahin, Anu Pejavara, Terrence O’Toole

**Affiliations:** 1 Centers for Disease Control and Prevention, Atlanta, Georgia

## Abstract

The burden of obesity and other chronic diseases negatively affects the nation’s health, businesses, economy, and military readiness. The prevalence is higher in certain geographic locations. Beginning in 2014, the Centers for Disease Control and Prevention’s Division of Nutrition, Physical Activity, and Obesity awarded funding to 11 land-grant universities through the High Obesity Program. This program implemented evidence- and practice-based strategies with a goal to increase access to nutritious foods and places to be physically active in counties in which the prevalence of obesity among adults was more than 40%. In these counties, funded land-grant universities developed partnerships and collaborations to work with community organizations, public health agencies, and other stakeholders to promote policy and environmental changes that address obesity. Data were collected by the Cooperative Extension Service in each selected county with technical assistance from land-grand universities and the Centers for Disease Control and Prevention. More than 2 million people were reached by the nutrition and physical activity policy, systems, and environmental interventions implemented.

**Summary**

**What is already known on this topic?**

Evidence is growing that strategies to improve physical activity and nutrition should focus on community-based approaches to improve health, especially in rural communities.

**What is added by this report?**

The High Obesity Program helped to increase access to healthier foods for more than 1.5 million people and increase access to physical activity for nearly 1.6 million people. More than 100 communities implemented policy, systems, and environmental changes that enhanced places for physical activity, and 88 priority communities increased access to healthier foods.

**What are the implications for public health practice?**

Public health strategies aiming to improve healthy food and physical activity access should consider working with nontraditional partners and using community-based participatory approaches to engage communities.

## Background

Obesity is a major public health problem in the United States and is associated with numerous poor health outcomes such as heart disease, stroke, and type 2 diabetes (1). To prevent and reduce the prevalence of obesity, the Division of Nutrition, Physical Activity, and Obesity (DNPAO) at the Centers for Disease Control and Prevention (CDC) provides support to state and local health departments and their partners to monitor levels of obesity and its risk factors among populations, and to implement and evaluate evidence-based strategies to improve nutrition and physical activity environments. In 2014, under an initial congressional funding authorization of $4.7 million (increased to $9 million in 2016), DNPAO developed the cooperative agreement *Programs to Reduce Obesity in High Obesity Areas*, known as the High Obesity Program (HOP).

HOP is a pilot program that funded 11 land-grant universities (LGUs) from September 30, 2014, through September 29, 2018, in states with a least 1 county in which the prevalence of obesity among adults was more than 40% according to data from the 2013 Behavioral Risk Factor Surveillance System. The purpose of HOP was to implement evidence- and practice-based strategies to improve physical activity and nutrition, reduce obesity, and prevent or control diabetes, heart disease, and stroke. In 2014, HOP began by funding a cohort of 6 LGUs (Auburn University, South Dakota State University, Texas A & M University, University of Kentucky, University of Tennessee, and West Virginia University). In 2015, two more LGUs (Louisiana State University and University of Arkansas) were added, and in 2016, three additional LGUs (North Carolina State University, Purdue University, and University of Georgia) received HOP funding (Figure).

**Figure:**
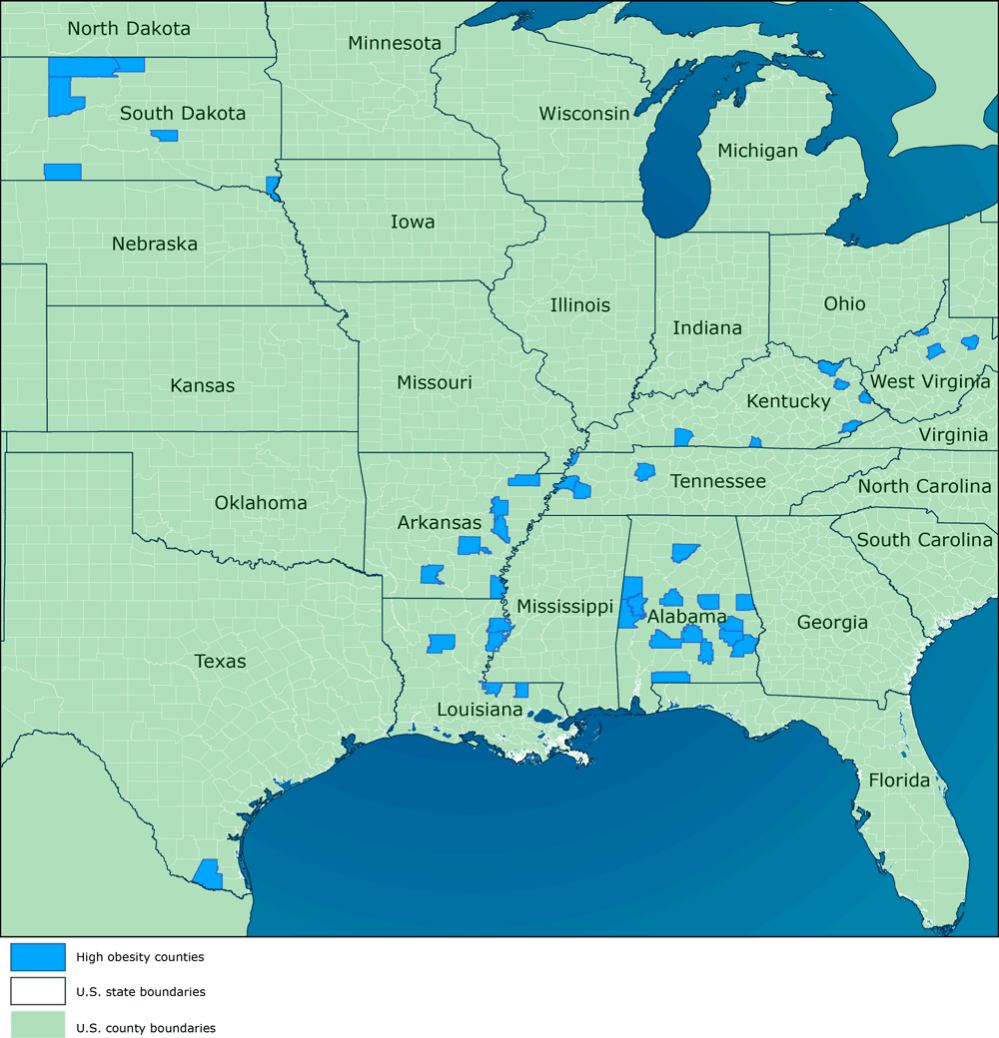
Counties selected for the Centers for Disease Control and Prevention’s High Obesity Program, 2014–2018. Sources: Esri (2), HERE (3), Garmin (4), Food and Agriculture Organization of the United Nations (5), National Oceanic and Atmospheric Administration (6), US Geological Survey (7), OpenStreetMap (8), and US Census Bureau (9).

**Table:** 

State	Counties
Alabama	Barbour, Bibb, Bullock, Chambers, Coosa, Crenshaw, Cullman, Escambia, Greene, Lowndes, Macon, Pickens, Sumter, Wilcox
Arkansas	Chicot, Craighead, Jefferson, Monroe, Ouachita, Woodruff
Georgia	Calhoun, Taliaferro
Indiana	Jackson, Lawrence
Kentucky	Clinton, Elliott, Letcher, Lewis, Logan, Martin
Louisiana	Madison, St. Helena, Tensas, Webster, West Feliciana, Winn
North Carolina	Edgecombe, Halifax, Lee, Northampton
South Dakota	Bennett, Buffalo, Campbell, Corson, Union, Ziebach
Tennessee	Haywood, Humphreys, Lake, Lauderdale
Texas	Hidalgo
West Virginia	Barbour, Gilmer, Pleasants

HOP was a new partnership approach to funding for DNPAO. The program provided an opportunity for DNPAO to collaborate with nontraditional public health partners — LGUs and their Cooperative Extension Service (CES) offices. DNPAO’s collaboration with LGUs and CES offices aligned with the US Department of Agriculture’s 2014 report, *Cooperative Extension’s National Framework for Health and Wellness*, which encouraged cooperative extensions to move beyond direct education efforts to increase knowledge and awareness and to focus on policy, systems, and environmental (PSE) strategies to prevent obesity (10). This redirection by CES resulted from growing evidence that social, economic, and environmental factors influence an individual’s health behaviors and outcomes (11,12). Consistent with the CES framework, through HOP, LGUs and CES were asked to expand their approach beyond direct education efforts to also focus on implementing evidence-based obesity prevention strategies with a focus on PSE approaches that facilitate healthy choices related to nutrition and physical activity in these high-risk, primarily rural communities. This article provides an overarching description of the intervention approach of HOP and highlights initial outcome data from the program.

## The High Obesity Program Approach

Given the growing evidence that community-based participatory approaches are effective in addressing health concerns in communities characterized by health disparities, particularly rural communities (11–13), HOP required CES to work with key stakeholders by engaging existing or developing new community coalitions to identify and support implementation of PSE approaches. The first year of the cooperative agreement was dedicated to stakeholder engagement and community planning. During this time, county extension agents helped to mobilize community coalitions. Coalition members included traditional public health partners as well as people representing a broad range of other organizations. Building and engaging community coalitions was a new approach to addressing obesity prevention, nutrition, and physical activity for CES. However, extension agents were thought to be well-suited for this role because, historically, they are often members of the communities they serve and have a deep understanding of local communities’ needs, context, and culture.

HOP recipients were required to implement interventions in 3 strategy areas in their selected communities (recipients could select multiple communities within a county). The strategy areas were 1) education and promotion; 2) nutrition; and 3) physical activity. Education and promotion strategies leveraged the strength and existing expertise of CES. Nutrition and physical activity strategies required that recipients extend their expertise and introduce PSE approaches to the communities in which they worked. Recipients could choose from community or early care and education settings to apply these strategies. Of the 11 recipients, only West Virginia University selected the early care and education setting. For the nutrition and physical activity strategy areas, recipients were required to select at least one intervention to address in their selected setting.

To better understand the communities that recipients selected for their HOP funding, the first year of the funding period focused on a community needs assessment and program planning activities. Extension agents engaged community coalitions at every stage of the intervention process, including during the needs assessment phase. Through this process, extension agents and coalition members gained a detailed understanding of community needs and assets, which helped in selecting, designing, and implementing interventions. The needs assessments often served as baseline information for LGUs and usually combined qualitative and quantitative data. After conducting needs assessments, the results were shared with community stakeholders. LGUs worked with coalitions to select priority areas and their corresponding interventions. LGUs encouraged coalitions to select topic areas where policy and environmental change would be feasible within the funding period.

LGUs also provided assistance to extension agents to build their capacity to implement HOP strategies in the selected counties. LGUs provided routine training and technical assistance calls with county extension agents. They established systems to support agents in data collection, reporting, and performance monitoring. Extension agents provided direct support to the counties they served.

To support recipients’ efforts, a 2-pronged approach was used, whereby CDC provided collaborative technical assistance to LGUs, and LGUs provided direct support to extension agents at the county level. As a part of its program infrastructure, CDC assigned project officers and evaluators to support each LGU during the HOP funding period. These CDC staff members have expertise in HOP program areas and provided technical assistance to LGUs on evidence-based nutrition and physical activity interventions, community-based participatory approaches, community needs assessments, and coalition development.

To monitor LGU progress, CDC, in collaboration with recipients, developed methods and metrics that recipients were required to report annually across 3 overarching data sources. The primary focus of data reporting was annual recipient updates on CDC-established performance measures (short-term outcomes) associated with each strategy. In addition, during the first year of the program, recipients were asked to report on community gaps and assets as determined by the needs assessments. LGUs also provided data on the intervention implemented in priority communities, including the counties in which interventions were implemented and the potential reach as determined by US Census data estimates. Lastly, LGUs provided detailed information to CDC on the resources (eg, financial, in-kind donations, volunteer hours, additional grant funding) they leveraged to support HOP-funded strategies.

The primary method for programmatic support and guidance occurred during monthly calls with each LGU staff member and their assigned CDC project officer and evaluator. Additionally, CDC evaluators facilitated monthly group calls with all LGU evaluators, which served as a forum for peer-to-peer learning on evaluation-focused topics. These calls, known as community-of-practice calls, also created an opportunity for recipients to provide CDC evaluators with feedback on reporting guidance for evaluation deliverables, including annual evaluation reports and performance measures. By engaging LGUs and soliciting their feedback, CDC was able to continuously improve technical assistance, guidance, and resources provided to HOP recipients.

## Program Outcomes From CDC Annual Reporting

The LGUs achieved outcomes across the PSE strategies they implemented. LGUs worked with 54 primarily rural counties, with a total population of 2,003,147. In total, 124 coalitions were engaged during the program period (2014–2018). Coalitions worked closely with key partners such as state and local health departments, local businesses, faith-based organizations, departments of agriculture and local agriculture offices, departments of transportation, school systems, law enforcement, and farmers markets.

The 11 LGUs were required to select at least one intervention under the nutrition and physical activity strategy areas (Table). Recipients were asked to track and report the number and type of PSE changes made and identify the priority communities in which interventions took place. CDC then used 2018 US Census estimates of the resident population data to accurately aggregate populations (14). Through HOP, LGU recipients increased access to healthier foods for more than 1.5 million people and increased access to physical activity for nearly 1.6 million people. Across the 11 funded LGUs, more than 100 communities implemented PSE changes that enhanced places for physical activity, and 88 priority communities increased access to healthier foods.

**Table:** Number of Land-Grant Universities (LGUs) Selecting Interventions and Population Catchment Area, the High Obesity Program, 2014–2018^a^

Intervention	Population Catchment Area by Intervention^b^	Number of LGUs Selecting the Intervention
**Education and promotion: provide education and promotional support for environmental approaches (implement both):**		
Outreach to children, adolescents, and families to increase healthy behaviors	2,003,147	11
Partner with community coalitions that support nutrition and physical activity		11
**Nutrition: implement evidence- or practice-based strategies to increase consumption of healthy food and beverages (select one)**		
Implement food-service guidelines and nutrition standards (including sodium) where foods and beverages are available	1,564, 631	6
Increase access to and promote healthy food at retail outlets		10
**Physical activity: implement evidence- or practice-based strategies to increase opportunities for physical activity (select one)**		
Create or enhance and promote access to safe places for physical activity	1,593,110	10
Promote joint-use agreements		6
Implement and promote Safe Routes to School or other walk/bike-to-school programs		4
Promote Complete Streets or other safe streets/community design initiatives		3

a Beginning in 2014, the Centers for Disease Control and Prevention’s Division of Nutrition, Physical Activity, and Obesity awarded funding to 11 LGUs through the High Obesity Program. The program implemented evidence- and practice-based strategies with a goal to increase access to nutritious foods and places to be physically active in counties in which the prevalence of obesity among adults is greater than 40%.

b Data source: US Census Bureau (14).

LGUs also identified and reported HOP-leveraged resources to CDC. Categories (eg, partner contributions, supplemental funding) and estimates for leveraged resources were developed by CDC by combining existing guidance with recipient feedback. During the final 2 years of funding, 2017 and 2018, LGUs leveraged more than $7.5 million across all reported sources.

## Implications for Public Health Practice

The design and implementation of this program has several implications for public health practice. First, CDC supported LGUs to work with local CES offices to implement evidence-based strategies to promote obesity prevention in community or early care and education settings. Through this new CDC collaboration, CDC identified opportunities to address obesity prevention via partnerships, stakeholder engagement, and nutrition and physical activity strategies within HOP’s community and rural context. For example, LGUs and CES were identified as fitting partners for HOP programmatic efforts because of their direct engagement with communities. CDC worked with LGUs and extension agents to leverage these existing relationships and engage with communities on community-driven needs assessments and strategy implementation. Additionally, the HOP technical assistance structure and collaborative approach between CDC and LGUs and between LGUs and CES at the community level provided a cohesive environment for clear communication, problem solving, and idea sharing to advance HOP strategies in communities. Other CDC programs or public health organizations may find HOP’s programmatic model optimal when working in a local community or rural context.

Second, HOP’s use of a community-based participatory approach supported community engagement and buy-in for strategy implementation and HOP program efforts. For example, the community needs assessment, which engaged community coalitions and members, helped to focus interventions locally by incorporating community knowledge and context into assessments and ultimately into interventions. As a result, HOP increased access to healthier foods and physical activity via PSE interventions in 54 primarily rural counties across 11 states. Local knowledge is essential for PSE change, and a community-based participatory approach may help strengthen the commitment from communities and increase opportunities for community support and sustainability. CDC programs and other public health organizations may consider this approach for potential programs.

Third, recipients leveraged resources totaling more than $7.5 million during the final 2 years of HOP. That HOP recipients were able to leverage resources from diverse sources (eg, partner contributions, volunteer hours, supplemental funding) is important. It may suggest that the HOP model is sustainable through its ability to acquire additional resources and engage additional partners and volunteers (15).

This brief evaluation of the HOP intervention has several limitations. First, the 2018 US Census estimates of the resident population reflected the population of priority communities in which interventions occurred, but anecdotal evidence suggests that, in some areas, residents from neighboring communities may have also accessed places to be physically active or to purchase healthier foods. Thus, the reach of the interventions may be underestimated. Second, because of the small population size of HOP priority communities, application of the interventions may be limited in their generalizability to the larger US population, particularly in urban areas. Third, funding periods differed by recipient. These differences may have limited the intervention scope and impact in some communities and produced different results among the 3 HOP cohorts. Lastly, CDC did not provide detailed guidance on funds leveraged until the final 2 years of the cooperative agreement. Thus, the total funds leveraged by recipients may be underreported.

The approaches described in this article provide an opportunity for public health organizations and CES to change community nutrition and physical activity environments to support obesity prevention.
